# Reviewing Bone Marrow Edema in Athletes: A Difficult Diagnostic and Clinical Approach

**DOI:** 10.3390/medicina57111143

**Published:** 2021-10-22

**Authors:** Umberto Tarantino, Chiara Greggi, Ida Cariati, Guglielmo Manenti, Matteo Primavera, Paolo Ferrante, Riccardo Iundusi, Elena Gasbarra, Andrea Gatti

**Affiliations:** 1 Department of Clinical Sciences and Translational Medicine, “Tor Vergata” University of Rome, Via Montpellier 1, 00133 Rome, Italy; chiara.greggi@gmail.com (C.G.); ida.cariati@uniroma2.it (I.C.); 2 Department of Orthopaedics and Traumatology, “Policlinico Tor Vergata” Foundation, Viale Oxford 81, 00133 Rome, Italy; matteo.primavera91@gmail.com (M.P.); riccardo.iundusi@uniroma2.it (R.I.); gasbarra@med.uniroma2.it (E.G.); gattiandrea14@gmail.com (A.G.); 3 Department of Diagnostic Imaging and Interventional Radiology, “Tor Vergata” University of Rome, Via Montpellier 1, 00133 Rome, Italy; gu.manenti@gmail.com (G.M.); paolo.abc26@gmail.com (P.F.)

**Keywords:** bone marrow edema, magnetic resonance imaging, athletes, joint overuse, pain

## Abstract

Bone marrow edema (BME) is defined as an area of low signal intensity on T1-weighted (T1W) MRI images and associated with intermediate or high signal intensity findings on T2-weighted (T2W) MRI images. BME represents a typical imaging finding that characterizes common stress-related bone injuries of professional and amateur athletes. The etiology of stress-related injuries is influenced by numerous factors, including the initiation of a new sports activity or changes in an existing training protocol. The clinical significance of BME remains unclear. However, a correlation between the imaging pattern of BME, the clinical history of the patient and the type of sports activity practiced is essential for correct diagnosis and adequate therapeutic treatment. It is also important to clarify whether there is a specific threshold beyond which exercise can adversely affect the bone remodeling process, as the clinical picture may degenerate into the presence of BME, pain and, in the most severe cases, bone loss. In our review, we summarize the current knowledge on the etiopathogenesis and treatment options for BME and highlight the main aspects that make it difficult to formulate a correct diagnosis and establish an adequate therapeutic treatment.

## 1. Introduction

Bone marrow edema (BME) is a nonspecific finding with multiple etiologies, defined as an area of low signal intensity on Tl-weighted (T1W) and high signal intensity findings on T2-weighted (T2W) on magnetic resonance imaging (MRI) [1]. Although on MRI what is detected is true local edema, histological evaluations have shown that lymphocyte infiltrates, fibrous tissue, increased vascularization, and decreased bone mineralization are also present. For this reason, BME has only recently been placed in the more generic and inclusive context of bone marrow lesions (BML), a heterogeneous clinical picture including lesions of the osteochondral unit [2]. The increase of water content in the bone marrow stroma, in association with a high density of blood vessels, seems to be the cause of the appearance of the signal detected on MRI [2,3]. Among imaging techniques, dual-energy CT is commonly used to map BME [4,5]. However, MRI is considered to be the gold standard for detecting bone marrow changes, guiding the decision-making process [6,7].

**Figure 1:**
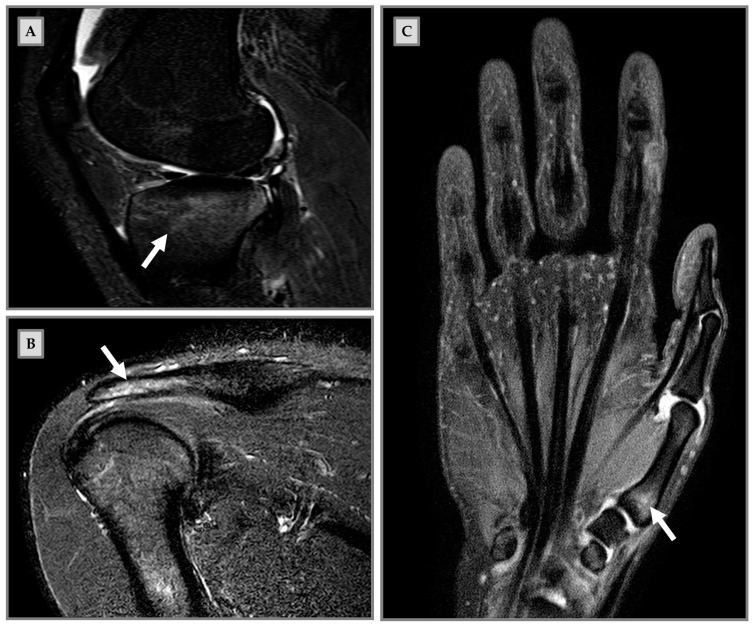
Bone marrow edema (BME) on magnetic resonance imaging (MRI) in clinical cases of nonprofessional athletes. (**A**) Male patient, 18-years old, art martial player. The patient had been in pain for three weeks during sports activities, elicited by kicking in the air. MRI sagittal short-tau inversion-recovery (STIR) shows BME (arrow) at the tibial plate. (**B**) Male patient, 20 years old, padel player. One-month pain and hypersensitivity during sports activity; he continued to train until functional impairment development. MRI coronal STIR shows BME (arrow) at the humerus diaphysis and the distal acromion end. (**C**) Female patient, 29 years old, volleyball player. The patient resumed intense physical activity after a period of no training. She presented pain onset during sports activity and functional impairment. MRI coronal STIR shows BME (arrow) at the first metacarpal bone.

**Figure 2:**
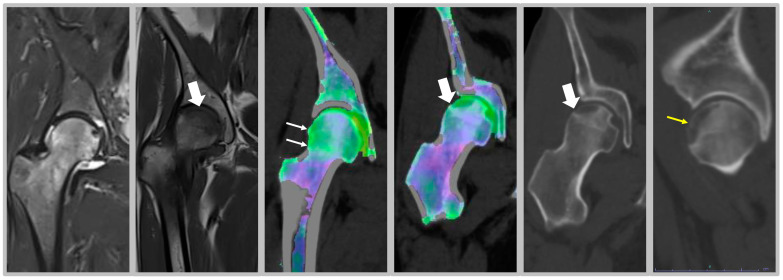
Clinical cases of nonprofessional runner. Female nonprofessional runner, 38 years old. MRI images in STIR show transient edema; T1 images (thick arrow) show small irregularity of cortical bone profile. Dual energy CT confirms the edematous pattern of the right femoral head, with BME appearing green against a purple background of normal bone (thin arrow). However, the edema appears more conspicuous in the subchondral area, where a large hypodense area appears on the partition images, with a small subchondral fracture well confirmed on the coronal (thick arrow) and sagittal (yellow arrow) reconstructions.

From a clinical point of view, MRI underlines the presence of BME in both symptomatic and asymptomatic subjects, particularly in high-level athletes and military recruits [8]. However, considering the significant importance of physical exercise in ensuring a healthy lifestyle, this condition extends to the population of nonprofessional athletes as well (Figure 1). Indeed, changes in an existing training protocol, equipment used, or the start of a new sport, especially in nonprofessional athletes, are frequent causes of stress injuries [9]. In Figure 2, we show the clinical case of a 38-year-old woman runner suffering from inguinal pain. Transient BME is suspected on MRI in STIR sequence, but T1 images show a small irregularity of the cortical bone profile. Dual-energy CT confirmed the edematous pattern of the right femoral head. However, the edema appears more conspicuous in the subchondral area.

In addition, the etiology of the injury may depend on a combination of the type of training, the playing load and intrinsic/extrinsic risk factors [10].

Biomechanical overload of joints and joint overuse can be caused by physical activity and intensive training, even in the absence of trauma, which also leads to the occurrence of BME in athletes. However, it has been suggested by some authors that the appearance of BME on MRI may also occur during the physiological process of bone remodeling as a consequence of regular overloading of the joint [11,12]. In particular, it has been hypothesized that in athletes, repetitive mechanical loading and an insufficient period of functional recovery may lead to joint overload and thus determine the nontraumatic onset of BME. In addition, this phenomenon may induce a reaction from the bone, known as a stress reaction, usually interpreted by the clinician as a prefracture [10]. In turn, the prefracture may evolve into a stress fracture in both normal and damaged bone [13]. Finally, there are contradictory studies in the literature regarding the hypothesis that the pattern of BME detected by MRI may be due to biomechanical aspects of the specific sports activity practiced [8,9].

Based on this evidence, the aim of our review was to (i) summarize the current knowledge on the etiopathogenesis of BME and (ii) highlight the main aspects that make it difficult to formulate a correct diagnosis and establish an adequate therapeutic treatment.

## 2. Literature Search Strategy

For this narrative review, 38 articles were selected from an initial number of 100 articles. The search was performed using the bibliographic databases Medline (1945, start date; 2021) and Pubmed (1916, start date; 2021). The search strategy was based on the use and/or combination of the following keywords: “bone marrow edema”, “athletes”, “sport”, “physical activity”, “joint”, “magnetic resonance imaging”, “bone marrow injury”, “diagnosis”, and “treatment”.

This narrative review included articles on the etiology, diagnosis, and treatment of BME in professional and amateur athletes. We considered studies that met the following inclusion criteria: (1) English language; (2) diagnosis established through clinical and MRI findings indicative of BME; (3) studies that included outcomes such as pain resolution, functional recovery, and time to return to sporting activity.

Studies were excluded if the patients considered had BME secondary to trauma, tumor, avascular necrosis, osteoarthritis, or infection.

The search process was performed on a worldwide basis, without excluding specific geographical areas or different ethnic groups. Language and species filters were applied to the results list to eliminate non-English articles.

## 3. BME Etiopathogenesis and Altered Bone Remodeling

Conflicting data are currently present in the literature about the causes of BME detection on MRI. Usually, a pure acute traumatic etiology is the main cause of BME in athletes, although in some cases this condition may also result from repetitive or chronic trauma [12]. As mentioned above, repeated stress triggers a stress reaction in the bone, which may result in hypertrophy and trabecular remodeling. Indeed, trabeculae subjected to this forced process exhibit microfractures, accompanied by the presence of BME on MRI. Mild trauma causes the appearance of BME without detectable damage to the cellular elements, whereas more severe trauma causes the appearance of microfractures and hemorrhage in the trabecular bone [10,12,14].

Biologically, bone responds to repetitive stress with an imbalance between the activity of osteoclasts and osteoblasts, which causes an alteration in bone turnover and therefore a weakening of the bone itself. Initially, the bone adapts by forming new periosteal bone to provide structural support. If the source of stress persists, there is a significant increase in osteoclastic activity to the detriment of osteoblastic activity, resulting in microfractures [10].

An interesting study by Matheny and colleagues demonstrated that joint overload leads to increased bone remodeling in regions affected by BME. Using a rabbit model, it was shown that microdamage induced by mechanical overload in epiphyseal bone results in enhanced bone remodeling and the appearance of BME within 1–2 weeks. In cortical bone, the generation of tissue microdamage appears to cause apoptosis of osteocytes in the immediate vicinity of the injury. This event, in turn, leads to increased receptor activator of nuclear factor kappa-Β ligand (RANKL) expression in osteocytes surrounding the region of injury, with consequent increase of bone resorption and remodeling [15]. The authors concluded that BME occurrence can be preceded by bone physiology changes, thus representing a potential target for preventive treatment strategies.

## 4. BME in Symptomatic and Asymptomatic Athletes

Numerous scientific studies have revealed that BME can be found in symptomatic athletes (Table 1). Symptoms are nonspecific but include the onset of pain, which is initially tolerable and not affecting the performance of physical activity, and tenderness during exercise. In this phase, the athlete continues to train, applying more stress to the joint, causing a stress reaction that can degenerate into a stress fracture [9].

To date, it remains unclear whether there is a significant correlation between BME and clinical symptoms, as the present studies are conflicting.

To validate the most reliable method for early detection of lumbar bone stress injury, Sims et al. analyzed the lumbar vertebral bodies of 65 healthy cricket players who reported lumbar symptoms and lumbar stress injuries. Their results showed that symptomatic relevant BME in the vertebral body was associated with a signal intensity ≥2. This suggests the existence of a potential correlation between symptomatic lumbar stress fractures and BME signal intensity [7].

In contrast, Paajanen and colleagues, through a 2-year follow-up study of 102 elite players (football, ice hockey, and bandy), found no significant correlation between the occurrence of BME and groin pain, as pubic joint BME was detected by MRI in 50% of both symptomatic and asymptomatic players [16]. Similarly, Varkas et al. analyzed the occurrence of BME of the sacroiliac joint in 22 military recruits before and after 6 weeks of intense standardized physical training. The evaluation after this time showed no statistically significant relationship between back pain and the occurrence of BME and no increase in its size [17].

According to some authors, the occurrence of BME can also occur in asymptomatic athletes (Table 1) [8]. For example, Major and Helms evaluated MRI changes in the knee joint of high-level collegiate basketball players before the start of the season that could have been misinterpreted as abnormal during the season [18]. The authors showed that 14 of the 34 players included in the study had BME in at least one location, concluding that the changes observed on MRI were asymptomatic abnormalities. Furthermore, it was hypothesized that microtrauma transmitted through the meniscus, dissipated by the cartilage, and absorbed by the bone, may be at the origin of these lesions. Finally, the continuous repetition of jumps and runs may have led to the appearance of BME [18].

The BME can be associated with any type of sports activity, which influences the localization of BME. In fact, Grampp and colleagues reported on a 29-year-old golfer with mild pain and swelling of the proximal phalanx II of her left hand. MRI revealed an involvement of the metacarpal joint and the proximal second phalanx of the left hand [19]. In agreement, Yochum and Barry reported the clinical case of a 42-year-old female jogger, who had persistent painful symptoms on the back of her foot, aggravated by the physical activity itself. Again, MRI showed involvement of the third tarsometatarsal joint characterized by the presence of stress-induced BME [20]. Moreover, rugby players show BME tending to the tibiofemoral joint (e.g., medial condyle), whereas runners show more involvement of the patellofemoral joint, due to more linear movement [21,22]. The different distribution of applied stress could also explain the different localization of BME and its lack of manifestation in some sites, such as the carpal bones [23]. Mandalia and colleagues, in a study of 25 asymptomatic university athletes, demonstrated a correlation between total training time, training intensity, and the occurrence of BME (*p* < 0.05) [22]. Based on their results, the authors suggest that the onset of BME might be particularly influenced by the intensity and duration of repeated impacts. Finally, that study confirms how the type of sport practiced is related to the incidence of BME, as 5 of the 13 rugby players showed a high incidence of BME, not detected in contrast while none of the swimmers, showing how the type of sporting activity dictates the incidence of BME [22].

Finally, some studies also suggest how BME size and its disappearance may be influenced by sports activity. In this regard, the study by Horga et al., in which the knees of 71 asymptomatic middle-aged athletes 6 months before and half a month after a marathon were evaluated, showed a reduction in the size of subchondral BME after the marathon for 19 of 58 subjects in whom the onset of BME had previously been detected [23]. Appearing and disappearing patterns were observed in a study by Kornaat and colleagues, in which 16 asymptomatic professional runners were followed for 7 months to study the clinical and radiological progression of BME [21]. At the beginning of the study, BME was purely localized in the foot and ankle joint in only 14 runners, whereas at the end of the evaluation period, 20% of BME appeared and 22% disappeared, showing a fluctuation pattern not associated with any clinical symptoms. The authors therefore concluded that BME may participate in the physiological process of bone remodeling, by not causing symptoms for at least the first 7 months after its onset [21].

## 5. BME Treatment Options

If left untreated, BME usually resolves spontaneously within 3–9 months. However, there are several treatment strategies, conservative or surgical, that aim to bring about a reduction in pain symptoms, when present, and accelerate the normal course of the condition [24]. Non-operative treatments of symptomatic BME include partial/non-weight-bearing, or the administration of pharmacological treatment, based on nonsteroidal anti-inflammatory drugs (NSAIDs), bisphosphonates and monoclonal antibody [25,26]. Müller et al. demonstrated that zoledronic acid can be recommended for relieving joint pain in athletes, and reduced recovery time by 50%, so that high-performance players can resume training [27,28]. Confirming the findings of Müller et al., Vasiliadis and colleagues demonstrated that the combination of a single dose of zoledronic acid with partial weight bearing for one month improves mobility and reduces BME [29]. In this study, 54 patients with bone marrow edema syndrome, who complained of prolonged pain and presented BME on MRI, were enrolled. At the 6-month follow-up visit, improved mobility was observed in 29 patients and resolution of BME was detected in 20 out of 54 patients [29].

On the other hand, ibandronate’s effectiveness has been shown in controlling pain symptoms in patients with knee BME. Küchler and colleagues report that a single intravenous administration of ibandronate leads to a reduction in pain in subjects with knee BME, regardless of the severity of the condition detected on MRI [30]. Among monoclonal antibodies, denosumab has proven to be the most efficient in the treatment of BME. Usually used for the treatment of osteoporosis, denosumab has also been shown to be effective in the treatment of bone marrow edema syndrome, as reported by Rolvien and colleagues [31]. In this study, 14 patients with idiopathic BME of the lower extremity were treated with a single dose of denosumab. After 6–12 weeks, MRI showed almost complete disappearance of BME in 93% of patients, while a complete recovery was observed in 50% of the individuals. Furthermore, visual analogue scale (VAS) evaluations revealed a clear decrease in pain perception. Finally, the serum dosage of some markers of bone metabolism showed a significant reduction in bone turnover after treatment [31].

According to the literature, teriparatide is used for the treatment of both fractures showing complications in the repair process and pathological conditions characterized by the presence of BME on MRI, such as complex regional pain syndrome I (CRPS I). Galluccio and colleagues, based on their clinical experience, argue that teriparatide is effective in the treatment of BME secondary to CRPS I, exerting a lasting effect in reducing pain symptoms and in the functional recovery of the joint. According to the authors, these effects may be due to the anabolic capacity of the drug, which is able to affect the cellular pathways of bone metabolism. They suggest a short-term period administration [32].

**Figure 3:**
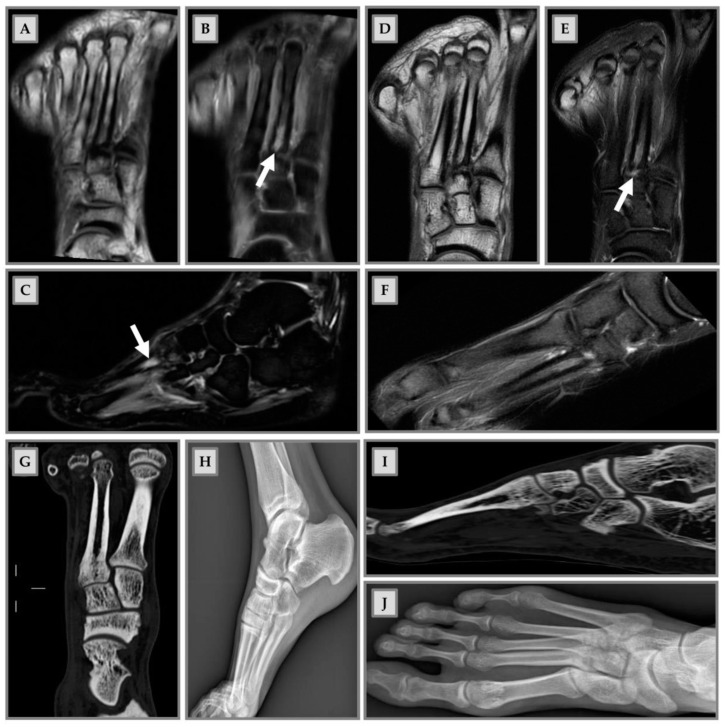
Male patient, 22 years old, professional rugby player. (**A**–**C**): Stress fracture of II metatarsal base; (**B**,**C**,**E**): altered signal intensity, hyperintense in short-tau inversion recovery (STIR), related to BME (arrows); (**D**–**F**): MRI check after 3 months: advanced fracture healing process and pain release; (**G**,**H**): CT check at 6 months after therapy. Fracture healing, pain release and sport restart; (**H**–**J**): radiography check at 9 months after therapy and 1 month of sports activity. Patient recovery.

Since BME occurs because of altered bone turnover, vitamin D administration has also been identified as an effective strategy for treating this condition, as it is a key element in maintaining the homeostasis of the bone microenvironment. In the study by Horas and colleagues, 31 subjects with BME of the foot and ankle were enrolled [33]. Among patients, a high rate of hypovitaminosis D was detected (mean value of 19.03 ng/mL). Consequently, the authors considered that an inadequate vitamin D level may be a cofactor in the onset of BME [33]. Capacitively coupled electrical field (CCEF) is one of the conservative treatments for BME. To investigate the role of CCEF stimulation, Piazzolla and colleagues enrolled 24 patients with acute vertebral compression fractures [34]. At 90-day follow-up, the group of patients treated with CCEF showed a reduction in vertebral edema and a significant improvement in VAS, compared to the untreated group [34]. In this regard, we propose in Figure 3 the clinical case of a professional rugby player, who came to our attention after a CT scan and MRI, which showed a stress fracture at the base of the II metatarsal of the right foot (Figure 3A–C); an altered signal intensity, hyperintense in short-tau inversion recovery (STIR) images, related to BME can be observed (Figure 3B,C,E). Initially, a pharmacological treatment based on the administration of calcium (1200 mg/day), vitamin D3 (800 I.U./day) and teriparatide (20 µg/day) was recommended, associated with CCEF. Control CT and MRI after three months of therapy showed an advanced healing process of the fracture; the patient also showed regression of painful symptoms (Figure 3D–F). CT scan after 6 months of therapy showed complete healing of the fracture with subsequent disappearance of symptoms and resumption of competitive sports activity (G and I). Plain radiography after 9 months of therapy showed complete recovery of the patient (H and J).

Operative treatments are considered if conservative treatment fails. Among them, core decompression and calcium phosphate bone substitute are included [25]. According to the literature, core decompression results in faster functional recovery, less chance of recurrence and better control of pain symptoms than patients treated with NSAIDs or paracetamol [35]. Calcium phosphate injection is a valuable technique by which fluid synthetic calcium phosphate is injected to fill the space between the trabeculae of spongy bone in the subchondral region, which is also referred to as subchondroplasty. Calcium phosphate mimics the strength and porosity of normal spongy bone, which will be used by osteoclasts and osteoblasts in the following months as a scaffold to remodel the local bone [24,36].

## 6. Discussion

BME can be considered a clinical picture with an unclear etiology, characterized by specific concerns. In the context of the athletic population, it appears that the primary cause of the occurrence of BME is not actual trauma, but repeated mechanical overload over time, which triggers a stress reaction from the bone. Under physiological conditions, the bone remodeling process is triggered by high intensity exercise [10]. Beyond a certain threshold, the remodeling process is not completed, resulting in an imbalance between bone apposition and resorption and alteration of the normal architecture of trabecular bone. In our opinion, this is when the stress reaction progresses to a stress fracture (depending on location), which on MRI appears to be characterized by BME [13]. The resulting imbalanced bone turnover may also trigger an inflammatory cascade, which only creates the necessary conditions for BME to begin.

Another issue associated with BME is its appearance on MRI in both symptomatic and asymptomatic subjects, suggesting that there are no warning signs that can predict the onset of this condition. Therefore, for the purpose of understanding the etiopathogenesis of BME, it is essential for the clinician to trace the patient’s medical history. When present, symptoms include the onset of pain and tenderness during sports activity. The characterization of the pain must be carefully controlled: its intensity, acute onset or chronicity, and its correlation with physical therapy must be recorded [8,37].

Because the symptoms are not specific, the athlete usually tends to underestimate them and continue training, causing repeated mechanical overload, which in turn leads to stress fracture and the appearance of BME on MRI. Therefore, it is essential to identify early signs of this type of condition to avoid incurring the fracture [9]. Based on our clinical experience, we can say with certainty that patients who regularly perform physical activity, at a competitive level or not, are characterized by a different ability to adapt to stress. The performance of an intense physical activity can have pathological outcomes or not, depending on the different adaptive response of the organism.

It is our opinion that this aspect could make it difficult to establish a standard protocol for the elaboration of a diagnosis of BME and, consequently, an adequate therapeutic treatment. There is currently no specific treatment for BME in athletes. Therefore, we strongly recommend that BME be treated in the context of everyone’s characteristics, with reference to mechanics, injury history, force load, and musculoskeletal maturation.

If joint mechanical overload due to physical activity is the primary cause of the occurrence of BME, the clinician should consider the intensity and mode of training, as well as periods of rest from exercise. In the absence of acute trauma but painful symptoms, the first approach is conservative treatment: immobilization for short periods, rest from training, and pharmacological treatments (such as NAIDs, bisphosphonates and monoclonal antibody) [25]. However, disrupting the injury dynamic with simple rest appears to be insufficient for optimal regression of the injury. In cases where conservative treatment fails, invasive strategies such as core decompression and subchondroplasty are used.

**Figure 4:**
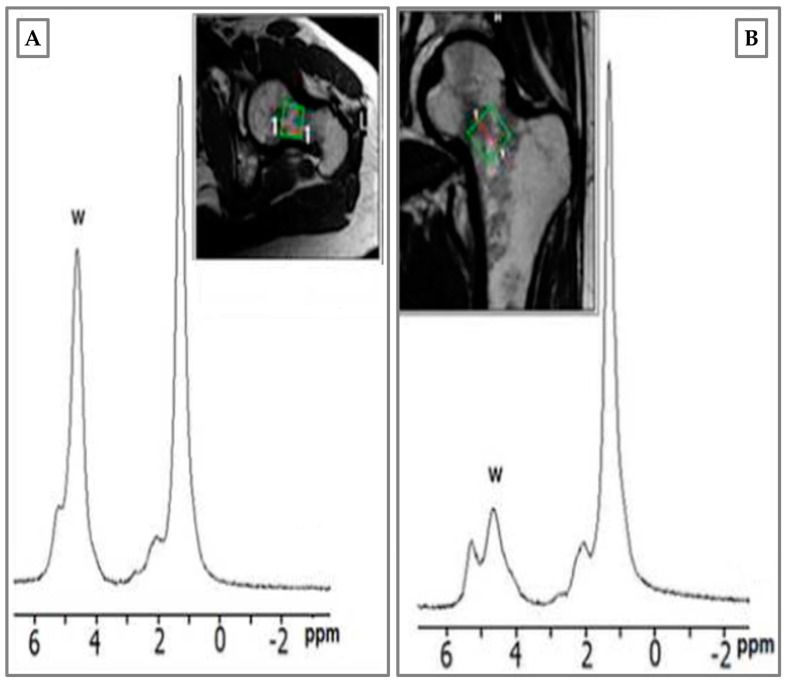
Magnetic resonance spectroscopy (MRS) of male patient, 34 years old, hockey player. BME of femur neck after tackling. (**A**) Water peak rising immediately after trauma; (**B**) BME disappearance at 3 months with water peak re-allineation to baseline. Green square: femoral neck area where the spectroscopic analysis was performed.

It is noteworthy that BME turns out to be accompanied by very small and difficult-to-detect lesions in the osteochondral unit. In this regard, MRI is confirmed as the most valid assessment tool for this type of phenomenon. In addition, the use of magnetic resonance spectroscopy (MRS) in the clinical workflow should be of interest in the field. As shown in Figure 4, MRS provides more accurate information about bone edema, and the subsequent presence of water immediately after trauma, and three months later. In agreement with reports in the literature, MRS has provided crucial information on bone composition based on the ratio of fat to water in osteoporosis [38]. Unfortunately, in our experience, MRS is not useful for the diagnosis of BME, but is important in the follow-up of patient recovery.

## 7. Conclusions

BME represents an aspecific clinical pattern with an unclear etiology that occurs in both symptomatic and asymptomatic subjects. In fact, studies in the literature show that the MRI-detectable pattern is not always accompanied by a well-defined symptomatology. Moreover, while some authors consider BME as part of the normal remodeling process, others consider it as bone abnormalities that should be treated.

Based on our experience, the clinician may therefore be faced with two types of situations: a BME found occasionally on MRI, unaccompanied by painful symptoms, and a BME detected following pain referral by the patient. In the first case, pain may or may not present over time; therefore, it is our opinion that the clinician should perform MRI to monitor the status of the BME and possibly reevaluate the patient’s clinical condition. In the second case, however, the clinical picture requires pharmacological treatment and surgical treatment in case of nonoperative treatments fail. Furthermore, since BME manifests with specific histologic features, such as inflammatory infiltrate, fibrous tissue, increased vascularity, and reduced bone mineralization, a histologic evaluation of BME could represent a predictive investigation of the patient outcome. This could represent a potential prognostic tool in more severe cases in which surgical treatment is used to resolve the clinical picture [2]. Finally, as the presence of BME is found both before and after the sports season, the assessment of BME by MRI could be used as a tool to monitor the health of high-performance athletes in Olympic programs. However, it is not clear whether the BME is the direct cause of the pain; thus, finding a possible causal connection should be addressed by future studies.

## 8. Limitations of This Review

BME is generally a clinical condition with a multifactorial and highly variable etiology. This makes it very difficult to make a correct diagnosis and to choose an appropriate treatment. In athletes, pain symptoms may or may not occur and the condition may therefore be detected on MRI at too late a stage, compromising the healing process. The limitations of our review are due to the small number of studies in the literature and their heterogeneity, as the results shown are related to different types of sporting activity and different associated BME patterns. Therefore, further studies are needed to better delineate this clinical picture in the athlete population, to facilitate the clinician’s diagnosis and in their choice of the most correct therapeutic treatment.

## Figures and Tables

**Table 1:** Studies carried out on the athletic population: main aspects of BME clinical picture in symptomatic and asymptomatic athletes.

	Reference	Participants	Age (Years)	Physical Activity/ Sport	Investigated Skeletal Segment/Joint	Follow-Up	Imaging Method	Results	Comments
Symptomatic BME	[7]	65 men	<17 <19	Bowling (cricket)	Posterior vertebral arch	8 months	MRI	BME signal intensity (2 or more) was positively associated with lumbar bone stress injury	BME MRI pattern is significantly correlated to symptomatology at the spine level
	[16]	102 men	>18	Soccer, ice-hockey, and bandy	Pelvis	2 years	MRI	No significant difference was found in the percentage of pubic BME between symptomatic players (8/15) and controls (20/43)	No direct correlation between the detection of BME on MRI and the perception of symptoms
	[17]	22 men	18–45	Standardized military recruits physical training	Sacroiliac joints	6 weeks	MRI	9/22 recruits (40.9%) already presented BME at MRI; these lesions did not increase significantly after 6 weeks of intensive physical training	BME size was not influenced by the performance of intense physical activity
Asymptomatic BME	[18]	12 men 5 women	18–22	Basketball	Knee	Preseason	MRI	14 (41%) of 34 knees showed BME in at least one location	BME on MRI may represent an asymptomatic abnormality; long-term evaluation is necessary to clarify the significance of these findings
	[22]	12 men 13 women	19–23	Football Rugby Running Netball Swimming Hockey Lacrosse	Knee	Postseason	MRI	7 participants (28%) were found to have BME (six in one knee and one bilaterally) 5 of 13 rugby players had BME, while none of the swimmers	The type of sport performed influenced BME incidence; the amount of training time, during the season, was significantly associated with BME appearance
	[23]	51 men 64 women	25–73	Marathon running	Knee	6 months before and half month the marathon	MRI	After the marathon, MRI showed a reduction in BME size in 19 of 58 previously detected BMEs	Physical training can influence BME size and disappearance
	[21]	13 men and 3 women	Mean age: 22.9 ± 2.7	Running	Pubic bones Hips Knee Ankle	Preseason and postseason	MRI	14 of the 16 athletes had BME lesions before the start of the season: 31/45 were in the ankle joint and foot; 26/45 fluctuated during the season, with new lesions occurring (9/45) and old lesions disappearing (10/45), without causing any symptoms	The observed fluctuation pattern could indicate that BME participate in the normal bone remodeling process and, within 7 months, does not cause symptoms

BME: bone marrow edema; MRI: magnetic resonance imaging.

## Data Availability

No new data were created or analyzed in this study. Data sharing is not applicable to this article.
